# Trust and reciprocity norms in the analysis of social capital related to udder health. A mixed methods approach with dairy farmers and veterinarians from the north of Antioquia

**DOI:** 10.1371/journal.pone.0277857

**Published:** 2023-11-17

**Authors:** Richard Zapata-Salas, José F. Guarín, Leonardo A. Ríos-Osorio

**Affiliations:** 1 School of Microbiology, University of Antioquia, Medellín, Antioquia, Colombia; 2 Research Group in Health and Sustainability, Research Group in Veterinary Microbiology, University of Antioquia, Medellín, Antioquia, Colombia; 3 Department of Agricultural Sciences, University of Antioquia, Medellín, Antioquia, Colombia; 4 Research Group in Agricultural Sciences–GRICA (Acronym in Spanish), University of Antioquia, Medellín, Antioquia, Colombia; Manaaki Whenua - Landcare Research, NEW ZEALAND

## Abstract

Understanding trust between dairy farmers and other actors in the dairy chain, as well as the reciprocity norms among them are fundamental to encouraging collective action and decision-making to improve milk production and udder health. The objective of this study was to understand the relationships of trust between dairy farmers and other actors in the dairy chain related to udder health in the north of Antioquia. Mixed methods (cross-sectional and grounded theory) with a convergent triangulation design were used. A total of 216 dairy farmers participated in the quantitative component, and 17 dairy farmers and 9 veterinarians in the qualitative component, they were located in 9 milk-producing municipalities in the north of Antioquia. A characterization survey of the dairy farmers, a survey on reliability and udder health, an analysis of each farm’s annual average BTSCC and CFU, and semi-structured interviews on the same topic were conducted. Problems were found in the biological indicators of udder health on the farms: BTSCC was poor for 67% of the dairy farmers, and CFU was poor for 22% of the dairy farmers. Veterinarians are the actors whom dairy farmers trust the most. Trust in dairy chain actors is complex, variable, and depends on many aspects. Trust representation, Socio-cultural Factors, Economic and Commercial Factors, Labor, Clinical and Laboratory Conditions of Milk, and Norms of Reciprocity constitute the categories of analysis when theorizing about trust and udder health. Each of the theoretical and emerging categories in this study describes actors, attitudes, behaviors, relationships between actors, and norms, allowing us to understand that trust between dairy farmers and other actors in the dairy chain in order to face the problems of udder health and milk production depends on technical processes, individual and collective human attitudes and behaviors, supply of services, political, regulative and economic determinants, the latter being transcendental in decision-making to invest in mastitis control and udder health care.

## Introduction

Milk is a priority item to guarantee food security, given its high nutritional value. In Colombia, 48.7% of the population consumes milk, of which 46% reaches the informal trade as raw milk [1]. There are few studies on the risk of milk production and commercialization for public health [1]. In 2013, it was found that only 0.7% of the milk analyzed met the criteria of total quality, the presence of pathogenic bacteria in milk, 5.5% of the milk positive to antibiotics [1]. In addition, it has been proposed that health risks to the milk consumer can originate in primary production when a series of biological, cultural, political, and economic conditions that affect udder health occur [1].

Udder health refers to the health-disease process in milk production systems with implications for productivity, economics, animal welfare, and public health. This dynamic and complex process is mediated by the human networks established between dairy farmers and the dairy industry actors. The concept of udder health can be understood within the categories of traditional epidemiology based on risk factors and disease; microbiology; genetics, resistance, and immunity; animal welfare; nutrition; organic production; cultural processes; and political processes. The latter two are transversal to all the other categories and the most researched theoretical developments in the last decade, with interest in the analysis of public policy in udder health [2].

One way of understanding political processes is through the theory of social capital. This study starts from this theory based on the expansionary view that establishes the relationship between social capital, collective action, and public policy. The theoretical proposal of Putnam et al. (1993) and Ostrom (2015), define social capital as "the value of trust generated by social networks to facilitate individual and group cooperation over shared interests and the organization of social institutions at different scales" [3].

Ahn and Ostrom (2002) propose three forms of social capital: (1) Trust and reciprocity norms, (2) networks and civil participation, and (3) formal and informal regulations or institutions [3, 4]. For the purpose of this research, this publication will focus on trust and reciprocity norms only. This study proposes an understanding of the phenomena of trust and reciprocity norms that are established between dairy farmers and other actors in the dairy industry about udder health. According to Ostrom and Walker (2003), trust is understood as "a specific level of subjective probability with which an agent evaluates that another agent or group of agents will perform a specific action." Therefore, trust allows the person trusting another to perform an action that involves the risk of loss if the trusted person does not perform the expected action. It also entails an opportunity for both the person trusting and the trusted person to increase their well-being. Reciprocity norms, on the other hand, refer to an internalized moral norm, as well as a pattern of social exchange in situations of collective actions [4].

The analysis of trust in dairy farming communities has provided insight into the importance of attitudes towards the science behind the advice, the credibility of the advisor or sources of information, the impact of the opinions and actions of other respected dairy farmers on decision making, and for udder health planning to improve milk quality [5–8].

In Colombia, there is no theoretical development on udder health as a political phenomenon mediated by the interactions between actors in the dairy chain. In this sense, dairy farmers and veterinarians are proposed and included as key actors and participants in the study to understand the phenomena of trust constructed, destroyed, and reconstructed between actors of the dairy industry around udder health and its consequences on milk production, commercialization, and public health. This study is based on a mixed methods methodological approach, conceptual from the theory of social capital and epistemological from pragmatism, assuming udder health as a complex phenomenon [2] whose objective was to understand the relationships of trust between dairy farmers and other actors in the dairy chain related to udder health in the north of Antioquia.

## Materials and methods

### Study design

Mixed Methods (Cross-sectional and Grounded Theory) with a convergent triangulation design in which results were interpreted by combining both types of data [9].

The epistemological basis of mixed methods is pragmatism, which supports the need for converging epistemological approaches to answer complex research questions. The use of mixed methods is justified by the need for understanding a phenomenon when neither approach is enough to tackle a research problem due to its complexity and the multiple dimensions of its reality [9, 10]. Data on the objective of the study could be collected at a given moment in time through a cross-sectional study. Meanwhile, the use of grounded theory allows for the deepening of theoretical categories and the recognition of emerging categories, i.e., new explanations, since it is not limited by a priori knowledge. In addition, its results allow the proposal of theories and concepts on subjects that have not been addressed very often.

### Study subjects

For the quantitative component, a non-probabilistic sampling stratified by municipality was carried out. There was a similar population of small, medium, and large dairy farmers who distributed their milk to processing companies, collection centers and/or the informal trade. In total, 216 dairy farmers, located homogeneously across nine municipalities in the north of Antioquia, volunteered for the study. The owner, manager, or head milker in charge of the production and with complete knowledge about the operation of the production system, and the supply and commercialization networks in each farm were identified and selected to participate.

For the qualitative component, two types of actors participated: dairy farmers and veterinarians working at the participants’ dairy farms. The number of participants was defined through theoretical sampling using category saturation [9]. The saturation of pre-established and emerging categories was reached with 17 dairy farmers. Dairy farmers were selected through maximum variation sampling aimed at detecting the multiple discourses typifying the human reality related to this study: gender, age, farm size, municipality, educational level, and roles at the farm. Dairy farmers were also classified as key actors, according to the results of the survey. Regarding the veterinarians, the saturation of pre-established and emerging categories was reached with nine of them. The maximum variation sampling included: gender, age, work experience at dairy farms, municipality, and employment relationship (employed at a milk collection/supply company or freelance worker).

### Inclusion and exclusion criteria

The participants of the study included owners, managers, or head milkers in charge of the production and with complete knowledge about the operation of the production system, and the supply and commercialization networks. Additionally, they had to be of legal age and located in one of the nine municipalities included in the study. Veterinarians working at different farms, some owned by dairy farmers across the nine municipalities chosen for the study, were also included after obtaining their consent and the data required for the study.

Individuals who could not attend any of the interviews or decided not to participate were excluded.

### Data collection

Two systematic reviews [2, 11] were conducted to define the categories of analysis for the surveys and the interview used in this study: 1. A survey with 9 questions to characterize dairy farmers. 2. A survey with 14 questions that assesses the agreement or disagreement with perceptions of trust between farmers and other actors in the context of udder health. 3. A semi-structured interview. An initial appearance validation and content validation of the items and topics of each instrument was performed with 4 experts in udder health, milk production, public health, and survey and interview design and validation. Subsequently, 40 subjects from the study population evaluated the preliminary surveys to determine their acceptability and applicability. An interview with categories, subcategories, questions, terms, and concepts was designed to find out the meanings and representations that dairy farmers and veterinarians relate to trust inside the network originating in the dairy chain and the government, in the context of udder health. The interview was conducted as follows: (a) Contextualization of the study and informed consent, (b) trust in improving hygienic quality and udder health indicators, (c) trust in veterinarians, dairy farmers, collection companies, and dairy chain, (d) cultural conditions of trust, (e) other structural conditioning factors of trust and (f) reciprocity norms. This outline only describes content since the interview was not conducted in a pre-established order. It was aimed at guiding the researchers so that the topics were discussed naturally during the interviews with dairy farmers and veterinarians. There were between two and three personal meetings with each participant, according to the open, axial, and selective coding format of the grounded theory [12]. Data was analyzed in-between interviews in order to tackle and reinforce unclear aspects from previous meetings.

Methodological rigor criteria of the qualitative component: The credibility, auditability, and transferability criteria were applied in the study [13]. Credibility was accomplished through lengthy discussions between the interviewer (“RZS” main researcher) and the participants, during which permanent confirmation of the findings was requested to gain an accurate insight into their realities. To guarantee auditability, two researchers made an independent coding (LAR and JFGM); a priori, it was determined that the final coding would originate in the consensus, and that, in the case of discrepancies, these would be forwarded to the third researcher. Regarding transferability, this study includes a sociodemographic characterization.

The data indicating sanitary, hygienic quality, and milk production were directly obtained from the milk companies to whom dairy farmers sell their milk, with dairy farmers’ prior authorizations. Laboratories are accredited with the norm NTC-ISO / IEC 17025: 2005. The averages for these variables were calculated by gathering quarterly data from September 2019 to August 2020. The variable CFU (colony-forming units) is presented in accordance with the ranges established by the Colombian norm and adapted by Múnera-Bedoya et al [14]: excellent (<75,000 units/mL), good (between 75,000 and 150,000 units/mL), acceptable (between 150,000 and 250,000 units/mL) and deficient (>250,000 units/mL). The ranges for BTSCC (bulk tank milk somatic cell count) were: excellent (<150,000 cells/mL), good (between 150,000 and 250,000 cells/mL), acceptable (between 250,000 and 400,000 cells/mL) and deficient (> 400,000 cells/mL).

### Analysis of the information

The categorical variables were described using absolute and relative frequencies. BTSCC and CFU were defined as dependent variables. The violation of the assumption of normality was verified through the Kolmogorov-Smirnov test with Lilliefors correction. Afterward, a Mann-Whitney U test was applied to evaluate the association between the variables in the characterization surveys of dairy farmers and the dependent variables. Then, linear regression was performed for the dependent variable CFU to assess whether the associations found in the bivariate analysis are mediated by confounding variables to the extent that the variables included in the model were those with a p value <0.05. The model was checked for the independence of residuals (Durbin Watson test), variance inflation factor VIF (collinearity diagnosis), homoscedasticity assumptions, histogram, normality plots, and scatter plot. SPSS software version 25.0 was used to complete the analysis and p <0.05 was considered statistically significant.

All interviews were recorded and transcribed with Transcribe software version 4.13.0, and then revised and corrected manually to guarantee complete accuracy. The transcriptions were read several times, which allowed the authors to familiarize themselves with the data. The transcriptions were later loaded into Atlas.ti software version 22. The interviews were analyzed in the three stages of open, axial, and selective coding. Open coding allowed a conceptualization based on the abstract representation of the phenomena described by the participants. In this regard, each text fragment was assigned a code signaling common characteristics and meanings. Such coding was based on theoretical categories pre-established by the authors and on the participants’ words. The axial coding was based on the codes created during the open coding. Categories and subcategories were established during this stage, as well as their relation in accordance with their properties and dimensions. During the selective coding, trust was determined as the central category and all other categories were integrated to propose a theoretical construct. The central category was defined in accordance with the following criteria, proposed by Strauss and Corbin: I. That all main categories are related to the central one; II. That each category or most of them provide indicators to the concept; III. That the relation between categories allows for a solid explanation; IV. That it explains the contradictory or alternative cases to the central idea of the category; V. That the concept is refined when it integrates others. The theoretical outline allowed for the elimination of the excess data and the completion of the categories still underdeveloped through additional theoretical sampling. The theory formed was validated by comparing it with the raw data and after the participants acknowledged the theoretical proposal as a close conceptualization of their realities.

The analysis of the integrated results was conducted in accordance with the mixed methods proposal with convergence triangulation design, through the comparison of similarities and integration of the qualitative and quantitative results in a matrix and the compared discussion of both paradigms [9].

### Ethical aspects

This study was approved by the bioethics committee of the SIU (University Research Headquarters) at Universidad de Antioquia, by means of act 19-101-876 and according to resolution 8430 of 1993 of the Colombian Ministry of Health, the Helsinki declaration, the Code of Federal Regulations, Title 45, Part 46 for the protection of human subjects, the departments of health and human services of the United States National Institutes of Health (1991), and resolution 2378 of 2008 of the Colombian Ministry of Social Protection. All participants provided written informed consent and authorizations to request milk volume, hygiene, health, and composition data from the milk companies where they sell their produce. The analysis was based on data that does not contain information conducive to identifying the participants.

Reflexivity statement: This study is based on our experiences in research and dialogue with dairy farmers where the analyses have led us to think that the problems of milk quality, udder health, general health and its relationship with public health, i.e. consumer health, are a social problem rather than a biomedical problem. This approach built with various actors invites us to consider udder health as a common good and its problems as social phenomena in need of interventions from collective action. In this context, the dairy farmer has been recognized as an actor in which responsibilities fall, and a fundamental actor to make interventions together with other actors. Based on the above, our epistemological and methodological position was born, and the instruments that allowed us to understand the phenomenon in its complexity were designed. From the methodological point of view, in this study, the authors have considered as many realities as people participating in the research, a theoretical sampling by saturation of categories and the selection of participants by a sampling of maximum variation with the aim of capturing the maximum plurality of discourses regarding our objects of study. The criteria of methodological rigor: credibility, auditability and transferability; compliance with the inclusion and exclusion criteria; and the coding strategies described above promoted a reflexive analysis, coherent with the realities of the participants and in consensus among the authors. The discussion and conclusions set out our reflections on each category.

## Results

A total of 216 dairy farmers were included in the quantitative component of the study, most of them adults between 27 and 59 years old (77,8%), 6% were women, most of the individuals only completed elementary school (51,9%), the average number of children per producer was 2.3 ± 1.75, which contrasts with the average number of children per producer working at the farm, 0.26 ± 0.56 (See Table 1).

**Table 1:** Characteristics of dairy farmers.

		n	%
Age	Young adult (from 20 to 26 years old)	11	5.1
	Adult (from 27 to 59 years old)	168	77.8
	Older Adult (from 60 years old)	37	17.1
Sex	Female	13	6.0
	Male	203	94.0
Educational Level	No Formal Education	8	3.7
	Elementary	112	51.9
	Secondary	54	25.0
	University Students	1	0.5
	Technical	10	4.6
	Technological	6	2.8
	Professional	23	10.6
	Specialist	2	0.9
Municipality	Carolina del príncipe	24	11.1
	Briceño	24	11.1
	Don Matías	24	11.1
	San José de la Montaña—San Andrés de Cuerquia	24	11.1
	Entrerríos	24	11.1
	San Pedro de los Milagros	24	11.1
	Yarumal	24	11.1
	Santa Rosa de Osos	24	11.1
	Belmira	24	11.1
Socio-economic status	1	22	10.2
	2	107	49.5
	3	60	27.8
	4	18	8.3
	5	3	1.4
	6	6	2.8
Role in the production system	Milker	10	4.6
	Owner without any role in the farm	34	15.7
	Manager, no owner	8	3.7
	Manager, owner	44	20.4
	Manager, owner, distributor	9	4.2
	Manager, owner, milker, distributor	108	50.0
	Manager, owner, technical-veterinary assistant	3	1.4
Cooperative and/or livestock association member	Yes	95	44.0
	No	121	56.0
Size of the production system	Small-scale (Less than 1529 liters/week)	87	40.3
	Medium-scale (Between 1530 and 3822 liters/week)	71	32.9
	Large-scale (More than 3822 liters/week)	58	26.9

The average annual BTSCC, regarding milk sanitary quality and considered a biological indicator of udder health, showed poor quality in 67.6% of the farms. On the other hand, the hygienic quality of the milk, in terms of the average annual CFU, proved to be excellent in 54% of the production systems (See Table 2).

**Table 2:** Bulk milk quality according to BTSCC and CFU.

	BTSCC			CFU	
	n	%		n	%
Excellent. Less than 150,000	3	1.4	Excellent. Less than 75,000	117	54
Good. From 150,001 to 250,000	19	8.8	Good. 75,001 to 150,000	39	18
Acceptable. 250, 1 to 400,000	48	22.2	Acceptable. 150,1 to 250,000	12	6
Deficient. Greater than 400,000	146	67.6	Deficient. Greater than 250,000	48	22

According to the results, the actors who dairy farmers trust the most are veterinarians. Also, most of them trust the laboratory as the best alternative regarding antibiotics selection and that following the recommendations provided by ICA (Colombian Agriculture Institute) in the Guidelines to Good Milk Production Practice leads to improving udder health. As indicators of reciprocity, the results showed that most of the participants have a positive view towards cooperativism and towards avoiding informal trade with low-quality milk if other dairy farmers commit as well (See Table 3).

**Table 3:** Trust and reciprocity norms of dairy farmers regarding other dairy chain actors, in relation to BTSCC and CFU.

			Average BTSCC	Average CFU
		%	Median (IQR)	Median (IQR)
1. I trust veterinarians, animal husbandry specialists, or professionals that tend my cows, and their recommendations regarding mastitis control and prevention.	Disagree	9.7	563756 (438650–670480)	104155 (53099–303134)*
	Agree	90.3	490200 (365800–670400)	54000 (22166–169444)*
2. I trust that the best alternative to treat mastitis in cows should be selected taking into account lab results.	Disagree	5.6	428846 (259084–751055)	71208 (13261–143745)
	Agree	94.4	496315 (371638–670480)	60222 (22874–209624)
3. I trust in the recommendations of other dairy farmers given their knowledge about udder health care.	Disagree	28.7	464878 (373400–614822)	40083 (20556–104155)*
	Agree	71.3	562003 (365800–687727)	79000 (24461–274750)*
4. I trust in the knowledge shared by university researchers regarding udder health care.	Disagree	6.5	475603 (387937–810854)	51150 (14844–104155)
	Agree	93.5	506155 (367558–670480)	65011 (23066–212625)
5. I trust that the milkers follow good milk production practices rigorously during the milking process.	Disagree	22.7	538422 (409044–670480)	68022 (22682–233244)
	Agree	77.3	488813 (354733–670480)	56822 (22682–169444)
6. I trust in the support given by the distributors of the milk I produce.	Disagree	19.4	471948 (357127–654786)	68256 (18812–233244)
	Agree	80.6	520206 (368067–687727)	56211 (22682–169444)
7. I trust that by following the recommendations provided by ICA in the Guidelines to Good Milk Production Practice, I can reduce mastitis cases on my farm.	Disagree	12.0	475603 (386400–675466)	91290 (38689–288044)
	Agree	88.0	501815 (365800–670480)	56199 (22166–183562)
8. I trust in the possibility of participating in the reconstruction of policies, norms, and regulations that help the milk producer in udder health care.	Disagree	7.4	506556 (410974–655544)	78911 (30511–164533)
	Agree	92.6	495123 (364887–670480)	54893 (22194–209624)
9. I trust in the support of the ICA representatives regarding udder health care.	Disagree	58.8	471666 (308600–670480)*	41044 (18640–139133)*
	Agree	41.2	635222 (399844–720089)*	84133 (38000–274750)*
10. One form of compensation for the dairy companies to whom I sell my milk could be continuous training for dairy farmers on issues related to the protection of udder health.	Disagree	4.2	452470 (219933–670480)	89000 (34289–303134)
	Agree	95.8	495156 (369875–670480)	56822 (22166–201353)
11. It is the government’s responsibility to promote regulations on the protection of udder health, enabling the participation of the actors involved and acknowledging the needs of the territory.	Disagree	9.7	488813 (317471–670480)	40444 (24844–150489)
	Agree	90.3	495156 (369875–670480)	63622 (22682–206622)
12. If other dairy farmers agree to use antibiotics prescribed by vets after following the lab’s recommendation, I can do so too.	Disagree	5.6	640161 (410344–731467)	59167 (29633–105309)
	Agree	94.4	490882 (367813–670480)	60222 (22452–209624)
13. If other dairy farmers agree not to sell milk from cows with mastitis and antibiotics, I can do so too.	Disagree	1.9	392221 (251122–772310)	31767 (15936–70678)
	Agree	98.1	496315 (367813–670480)	65011 (22682–209624)
14. I consider that being a member of cooperatives or other organizations of dairy farmers is a good alternative to working as a team in udder health protection.	Disagree	8.3	514533 (297291–670480)	113838 (22682–169444)
	Agree	91.7	492986 (368067–670067)	56199 (22682–206622)

In the linear regression model, CFUs were explained in 4.1% by 1. Trust in the veterinarian and other professionals attending to the farmer’s cows; 2. Following the recommendations of other dairy farmers for udder health care (see Table 4).

**Table 4:** Linear regression model for CFU.

Dependent Variable	Model variables	value p	Regression coefficient	Determination coefficient
CFU	1. I trust the veterinarian, animal technician, or professionals who attend to my cows, in their suggestions for mastitis control and prevention.	0.023	-.153	4.1%
	3. I apply recommendations from other dairy farmers given their knowledge in udder health care.	0.033	.144	

### Categories and subcategories defined during the analysis of trust and reciprocity norms related to udder health

The qualitative analysis of the interviews with dairy farmers (DF) and veterinarians (V) led us to recognize six categories: Representation of trust, socio-cultural factors, economic and commercial factors, labor conditions, clinical and milk laboratory, and reciprocity norms.

#### I. Representation of trust

For dairy farmers, trust in the context of udder health represents first, self-confidence, understood as the capacity of believing in their abilities and experiences to solve udder health issues, as well as feeling the joy and pride that results from doing labor that was inherited. However, they acknowledge themselves as social subjects that need to trust in other actors, which is conditioned by their background. Usually, veterinarians are the actors with whom dairy farmers establish more vertical interactions.

*DF2*. *First of all, you must trust yourself; also, you trust trained people*.

For veterinarians, trust is one of the most necessary elements for collective action, they consider that trust between dairy farmers and them is represented by the expectation of that relationship leading to solutions to udder health issues. In this sense, dairy farmers’ trust is mediated by veterinarians’ knowledge, experience, and mainly, in the results of their interventions. For veterinarians, trust in dairy farmers is represented by their interest in discussing interventions and working as a team to make appropriate decisions. It is a way to acknowledge their culture and their ancestral knowledge. However, in some cases, excessive producer’s trust based on their experiences, without the support of a multifactorial analysis, surpasses medical criteria supported by scientific knowledge.

*V2*. *We think that they are people who simply ignore everything about this problem, but sometimes they know more out of experience, and sometimes they know things that we, as professionals, don’t even know*.

#### II. Socio-cultural factors of trust

In this category, we find a series of conditioning factors mediated by socio-cultural aspects that promote or hinder both construction and stability of primary farmers’ trust in the networks that they establish with other actors in the dairy chain to improve udder health.

For veterinarians, trust is a fragile social phenomenon between dairy farmers and other actors in the dairy industry that requires time, patience, and evidence based on knowledge and practice to achieve credibility. If there is credibility, there is trust. In this process, charisma and empathy are essential to work with dairy farmers.

*V1*. *there have been veterinarians that do nail it, technically it is, people who know a lot*, *who like to update themselves! And they do not tune in with dairy farmers or with the job, maybe because they are serious-looking or lack charisma or empathy to deal with people while having very good techniques*.

Many of the dairy farmers are highly demanding in what they consider indicators of trust, however, regarding the use of antibiotics and other medications, it seems that they are willing to accept recommendations from several other actors with or without knowledge on the subject.

*DF1*. *yes, maybe companies, and those in the agricultural stores who sell you medications, or many friends, like veterinarians and also neighbors who may have more experience or know more*.

*Trust-promoting socio-cultural factors*. For dairy farmers, continuous training on udder health gives them the self-confidence to promote udder health and thus healthy milk.

*DF1*. *When knowing more about it and learning more about the ropes, you are to improve*.

For dairy farmers and veterinarians, trust is built through a discussion based on theoretical foundations, experience, and respect in the communication process. Veterinarians strengthen their trust in dairy farmers when they intervene completely as planned to address issues affecting udder health.

*V3*. *When I have a client who does not pay attention to my recommendations, I prefer not to have him as a client so as not to suffer others’ mistakes. I’m not going to let others think I’m a bad vet because there are people who won’t do what’s necessary to have good production*.

Successful experiences of dairy farmers in controlling mastitis constitute a cultural behavior that favors trust among dairy farmers. It is possible to rely on the recommendations of other dairy farmers who have faced and solved similar problems successfully.

*DF14*. *Well, in any case, you’re always talking a lot with other dairy farmers and trying what others have done to see if it works for you*.

Proactive dairy farmers rely on themselves, their training, technology, and scientific knowledge to design plans that benefit udder health.

*DF14*. *Meaning that we’re going to talk about the subject of technology, obviously to help out a little; the subject of monitoring prevention of somatic cells, trying to do even a CMT once a month or fortnightly. We’re going to see how we manage to deal with this issue, udder health, that is, trying to classify animals well. What’s no longer really working because of the issue of somatic cells, we must rule it out. I think that’s how one should work*.

Professionally trained dairy farmers are much more critical. They design plans to ensure udder health planning and establish production, prevention, and control protocols. However, veterinarians remain as trusted key players when udder health issues worsen.

*DF16*. *As things develop. And when something about handling and protocols gets out of hand, then I must look for a veterinarian to help me*.

*Trust-hindering socio-cultural factors*. Dairy farmers find it very difficult to build trust when there are no common interests. All responsibility for udder health has fallen on dairy farmers. As primary links in the dairy chain, dairy farmers consider that the collaboration networks between actors of the dairy chain are not strong enough to face the challenges and difficulties of primary production.

*DF5*. *You have to deal with it on your own. What they offer you is very little. They only care about what’s theirs. A company barely sends someone to help. Everyone has to deal with it on their own*.

Dairy farmers consider that the lack of state control over milk collection companies, which are the ones who ultimately control the price of milk, discourages trust in undertaking programs to improve udder health, given that the dairy farmers consider that the payment for CFU and BTSCC is very low and susceptible to manipulation.

*DF4*. *Actually, there is no trust. We are as preventive as necessary, and when it comes to selling, we don’t have a good product, in reality, they always describe it as bad, we never offer a good product, so there is no trust*.

*DF1*. *The incentive is very little, and the company’s incentive for mastitis is very low, so people do not worry much.*

*DF10*. *A: Oh yeah. Trust is lost because if you see low bacteria and suddenly, they go up, then you say something must be wrong, either they are changing the sample or the results. Then, you no longer trust*.

There are neither norms nor regulations that promote the economic growth of the agricultural sector nor the dairy sector in particular. Thus, dairy farmers face economic difficulties that prevent them from investing in udder health planning, mastitis control and prevention, and therefore, public health and the country’s food security.

*DF4*. *The government is neglecting the largest food source in Colombia, which is the countryside. Not only the dairy products, because I was also a dairy farmer, and it didn’t go well in agriculture at all*.

Dairy farmers’ ancestral knowledge, culturally inherited, leads to excessive trust and variable mastitis intervention results.

*V1*. *but obviously there is overconfidence, many times there are bad decisions that threaten the animal’s as well as its well-being*.

The veterinary care that collection companies or input suppliers offer dairy farmers is deficient. They do not meet dairy farmers’ needs in terms of supply, frequency, and face-to-face assistance on the farm. These are all factors that hinder dairy farmers’ trust in veterinarians and in the companies of the dairy chain related commercially to them.

*V9*. *Most of them, I would believe that 90 percent are reproductive checkups… Consultations in udder health problems are six to seven percent, especially by phone*.

In this regard, dairy farmers consider that telephone assistance causes mistrust in veterinarians’ recommendations.

*DF1*. *We know better how the herd is doing because you talk to the veterinarian on the phone, but the veterinarian is not seeing the animal, instead, you are right next to it*.

Dairy farmers often prefer to hire private veterinarians rather than wait for the veterinary attention offered by the collection company, as these are not always available to attend promptly to the issues affecting udder health. The experience led, in many cases, to the loss of the quarter.

*DF13*. *We don’t wait. When we need a veterinarian, we pay for a private one*.

#### III. Economic and commercial factors

This emerging category comprises a description of some structural determinants experienced by dairy farmers that have indirectly conditioned milk production, udder health, and dairy farmers’ trust in collection companies, suppliers, the government, and veterinarians.

Free trade agreements impact dairy farmers in Colombia negatively. Trust in the government has been lost since it allows large quantities of lower-cost milk to enter the country, thus affecting the internal market. The consequences reflect in milk prices, the limits to milk volumes purchased from dairy farmers at certain times of the year, and the partial or complete decrease in the purchase of milk from the national producer “*enlechadas”*.

*DF1*. *It’s true that Colombia produces milk for the country, for the country’s internal consumption, and then the other comes in and they are going to lower the prices. Because it’s not the same, because here in Colombia we are charged for producing and in other countries they are paid to produce*.

*DF3*. *They say that the Government allows many products from other places to enter the country. That’s something that affects one a lot. That’s where the milk limit comes from, when there is already so much of other milk in the cheese, they take advantage*.

With the free trade agreements, large amounts of whey also enter the country. The collection and processing companies buy them, thus reducing the amount of milk purchased from Colombian primary farmers.

*DF4*. *How much trust is there going to be if the government itself is flooding us with whey out of the free trade agreements so that the companies fill their coffers and the poor producer… sits on pins and needles every day? They give away that whey because it is a by-product, it is waste for them, so they throw it away in countries where there are no controls*.

*DF13*.* Sure, imports are very cheap for them, that’s why they punish us*.

The trust relationship between the dairy farmer and the dairy companies is deteriorated by the price of milk. Dairy farmers have identified discrepancies in milk payments and lack of government control over the dairy industry. In addition, farmers face economic pressures due to the low annual increase in milk payments and high increases in the cost of production inputs.

*DF3*. *So, what do we dairy farmers do? We keep milking cows to death… and the price of milk is the same. Twenty years ago, it was the same, then, look how much more we’re being charged for our concentrate this year*.

*V4*. *I mean, trust between the dairy farmer and the milk company has been lost. There are discrepancies in the payment. Some dairy farmers are very thoughtful and are paid very well for their milk, but others are very thoughtful and aren’t paid well*.

In addition, corruption in economic support programs for food dairy farmers has deteriorated the trust between dairy farmers and the government.

*DF5*. *Support is given to dairy farmers, for roads, but where does it all go? It is distributed among themselves. You saw what happened with Agro Ingreso Seguro, for example… Where did all that money go? It was given to the rich, but the rich among them, and not to individuals*.

Veterinarians have pointed out other economic determinants of trust between the dairy farmer and the veterinarian with harmful outcomes in udder health. The cost of an integral treatment for mastitis can be high because it includes antibiotics for several days and anti-inflammatory drugs, among other medications. This situation influences the dairy farmers’ decision-making with consequences in the trust in the collaboration networks, production, udder health, and economy.

*V2*. *Sometimes you prescribe three or four things, and they only buy one, and then say that nothing worked! But in reality, the treatment is administered for one day only. They think that one day is enough. And for me, that is the worst possible case*.

*V6*. *They follow perhaps half of the recommendations, and then they ignore the rest, without a scientific or analytical reason, or sometimes they just don’t have the money to compensate for the contributions one makes to help them improve*.

Another element described by veterinarians is the demotivation of dairy farmers. They consider that the bonuses have always been an incentive to improve udder health and, therefore milk quality. They are concerned that bonuses have been gradually withdrawn, which may have generated a lack of motivation among dairy farmers, a mismatch in the final price of milk, low profitability, and, consequently, a decrease in investment in udder health care.

*V3*. *Bonuses used to be paid. And now they have been taken away, withdrawn. So, you have to keep producing without much help and you have to produce well so you don’t run out of animals and to be able to have a steady trade for your milk. But all those allowances have been progressively withdrawn*.

#### IV. Labor conditions

This category is developed into two subcategories; the first refers to the labor force hired on the farms and the problems in udder health due to the lack of motivation of dairy farmers to work in dairies. The second presents the reality of the relationship between the labor conditions of farm veterinarians and the quality of health services.

Trust between the dairy farmer and his workers has deteriorated significantly. Dairy farmers consider that hiring farm workers is a problem to improve udder health, regardless of whether it is permanent or one-day replacements. Dissatisfaction with labor conditions is common among workers, which manifests itself in a lack of commitment, disinterest in communicating udder problems observed, and high labor demands. Despite the situation, large production systems require more workers and may have, therefore, more udder health and animal welfare problems.

*DF6*. *A producer told us that he lost three workers in one week, that is to say, labor is a very complicated issue. What is happening? They want good salaries, not to work as hard, and they lack commitment. This is another cause of poor control of mastitis, it is not the same as my husband who cares and who got his cows with sacrifice and who loves them as his daughters, and it pains him to see them sick and he worries. A worker doesn’t say anything when he sees a sick cow, he milks in any way to finish quickly. He doesn’t follow the proper milking routine: the foreclipping, the pre-sealing, the udder management…all is rushed. So, the labor issue is on a tightrope. You hear stories about this worker who lasted three days or another worker who milked in the morning and then left, so changing workers so often also affects mastitis, everybody milks but does not milk the same*.

As a result of this situation, dairy farmers consider that the countryside is running out of labor. People are becoming more and more demanding to work in dairies.

*DF3*. *Look at what is happening now to find a replacement, even for one day, you cannot find anyone because people do not want to work on farms anymore*.

Large farmers try to motivate their workers with good labor conditions; some of them make them participate in a percentage of the bonuses received in the payment of the milk. They often rotate between farms according to the minor economic differences offered. Worker desertion forces a constant search for new workers for the farms.

*DF15*. *Most people accept work for economic reasons, where they get paid better. It is constant competition*.

*DF5*. *To have workers do things right, I offer them a percentage of the bonuses that I get from the milk*.

Labor conditions are not optimal for the veterinarians hired by the collection or input companies that provide technical assistance to the dairy farmers who buy their inputs. This situation means that many of the veterinarians who work on the farms are recent graduates coming to the countryside to gain experience. This is a short stage that ends abruptly once they have gained experience and received a better job offer. Veterinarians resign at a time when trust with dairy farmers is just being established, and communication has improved, causing the cycle to start once more with the entry of another professional.

*DF1*. *And so, a new person comes! And you must build that trust again*.

*V7*. *For starters, we have to know about the epidemiology in the area. Things are not the same in all areas, because of the culture, because of the herd that they have, because these are closed herds. After all, these are small municipalities, because you need to earn the dairy farmer’s trust, that is, you have to earn it first, by explaining the situation to him and second, by establishing a treatment that yields results so that he says, “If there are results, fine*.”

Veterinarians are overloaded in relation to the number of dairy farmers they have to attend. This generates a low frequency of attention, untimely attention, distrust, and the perception that the veterinarian is selective.

*DF9*. *There is a lack of monitoring. They say it themselves, around here vets only come once per year and very occasionally, or the vet is selective and only goes to the rich dairy farmers, not to the poor, not to where they only have three and four cows. They express it many times. But then, how can we strengthen it? More accompanying personnel is needed because the burden for only one professional is heavy; 200 dairy farmers is a lot of people*.

### V. Clinical and milk laboratory

The clinical laboratory service for mastitis analysis is considered by veterinarians a fundamental ally in achieving an appropriate diagnosis and treatment. This category comprises dairy farmers’ and veterinarians’ views on the relevance of the clinical and milk laboratory in building trust between dairy chain actors in the context of udder health.

The attitude of dairy farmers regarding not investing in laboratory diagnosis of mastitis cases negatively affects trust since working without diagnostic aids sometimes results in deaths, continuity of the disease in the animal, and resistance to antibiotics.

*V8*. *This obviously limits a vet to make a good diagnostic, so if the vet starts to order tests or says something a producer does not want to hear, this is not good, and the trust is lost. And so, the trust issue, I would say is closely connected to a social issue, to the ability of a producer to assess the challenge a veterinarian faces without diagnostic aids, and many times, without the necessary elements to get the right diagnosis or a good treatment*.

There are two reasons considered by dairy farmers for not using the clinical and milk laboratory. First, the remoteness of diagnostic laboratories generates disinterest in using this service; second, the long delivery time for laboratory results generates disinterest in using this diagnostic service, which has direct effects on decision-making in the appropriate use of antibiotics and bacterial resistance.

*DF17*. *I have some land in San José de la Montaña, and taking time to go to San Pedro, well it’s useless. It’s a waste of time. You get there in the afternoon*.

*DF17*. *They call me frequently because, on surveys, I say the good and the bad. I am always a very fair person, and the bad thing about them is that they are very slow. What is the purpose of sending a milk sample for CFU on Monday, for results to arrive next Monday? When I get the payment slip on Thursday, I did nothing. I mean, if I wanted to fix a problem, what is the purpose of sending that sample?*

On the other hand, dairy farmers see in the analysis of milk samples, which finally determines their payment, a conflict of interest from the collection companies. Only one of the collection companies performs the analysis of the milk collected in its own laboratory; the rest of the collection companies contract an external certified laboratory. Dairy farmers state that there is no coincidence between the results of the milk quality indicators provided by the company and the results provided by an external laboratory when they compare samples of the same milk. Regardless of whether the laboratory is internal or external, dairy farmers never receive the results directly from the laboratory; the results are only available on the invoice or payment slip for the milk, which has generated mistrust towards the collection company.

*DF12*. *So, you read there that milk has no fat, but if you test the externally, the milk has fat*.

#### VI. Reciprocity norms

The reciprocity norms refer to a pattern of social exchange manifested in situations of collective actions. It starts with the cooperative initiative and is strengthened to the extent that cooperation is reciprocal. This category was constructed based on the cooperative attitudes that favor or disfavor udder health described by dairy farmers and veterinarians.

Some veterinarians consider that there is trust between dairy farmers, they make recommendations to each other based on experience, and ancestral and cultural knowledge of the community. In many cases, these recommendations neglect medical recommendations. These cooperation processes have sometimes generated bad decisions, affecting animal welfare and the economy of the production system.

*DF12*. *I have many friends, and we share a lot of information that can help me when the time to make decisions comes and evaluate the best way to go*.

*V2*. *You know that is so discouraging! I believe all my colleagues you will get to interview have the same issue happen to them. Sometimes you recommend something, but then the neighbor comes and says, "I had the same situation, and I did this and this, and it worked." And some people have, excuse me, the gall to call and ask: "look, the neighbor told me it is better to do this other thing, what do you think?" And I already gave my opinion because I have already made an examination, and I bothered to go to the farm and leave a prescription, for the neighbor to come and say this other thing is better. In most cases, they listen to their neighbor*.

Leaders are considered by veterinarians as key actors to promote collective action through the strengthening of reciprocity norms, where a community of dairy farmers replicates good practices in pursuit of udder health when that leader performs and recommends it. However, there is little or no population of leaders in the villages, which represents a problem for collective action in the north of Antioquia.

*V8*. *Especially when we were in the municipal administration, we looked for support from these leaders, they are key players because they have credibility in the village. So, if they see this person doing these actions, well, others will want to do them as well. It sticks, it is by word-of-mouth, this training of trainers, which is what we call these extension processes, that you increase the technical skills in the village*.

Among dairy farmers, the contact, the interest in collaborating, in collective action has been disappearing. As described by veterinarians, this is a generational attitude that has become predominant in the last few decades. Nowadays, individualism prevails.

*V7*. *The generation that has had a large portion of the milk business in Antioquia has been disappearing. Old people, in the past, they were very close to each other, they were very supportive, of the neighborhood. Very fond of each other. But that is not happening anymore. Why? Because some people have educated themselves a little more, have had the opportunity to take a course or a technological program, and they keep everything for themselves. They don’t understand they have an obligation toward the community around them*.

*DF1*. *They don’t think about others, they just want to fill their own pockets. If everything is going well, my pocket is doing well. I won’t care much about my neighbor*.

Sharing a cooling tank is a practice that dairy farmers who cannot afford to buy a personal tank have agreed on, out of solidarity with a family member or friend, or for business purposes. This practice is based on reciprocity norms; however, in most cases, it has negative economic results for the dairy farmers of better-quality milk.

*DF15*. *A neighbor there pours about a hundred liters, he is her brother, because it makes me kind of angry, but it’s not fair. Someone with shared tanks has problems*.

The government it only expects to collect taxes, but there is no retribution to communities of dairy farmers neither support by the ICA, nor policies or programs that favor udder health care and producer growth. These particular government interests destabilize reciprocity and trust among parties.

*V3*. *We are nothing but a number for the government. If you pay UGPP, which is a parafiscal charge and all that stuff. You have to comply with all of this, you pay taxes on the farm, checked, withholding tax, checked. In other words, the government is more interested in what it receives from us than in giving something back to us*.

*DF5*. *The ICA is there just to screw us over! They just pay attention to brucellosis and tuberculosis, but they have no role and no commitment to dairy farmers regarding udder health*.

Although all dairy farmers recognize the impact that the sale of milk from cows with mastitis and antibiotics has on public health and express their attitude of not selling such milk to the informal trade, the economic crisis in which they find themselves has led them to act inconsistently.

*DF5*. *What is happening is that nobody wants to lose. And honestly, we don’t have the support of the Colombian government or any other institution, so we just try to survive. We must survive. We know that milk should not be sold but then, who helps us? For example, at this very moment, the milk price is not bad, I am not complaining about the price of milk, and most people are not complaining about that either. They complain about the high price of supplies, the fact that a bag of fertilizer is currently going up to 20,000 pesos more, the feed for livestock, the salt, the veterinary drugs, everything is so expensive. So, what do we do? We try to survive somehow. And some people… the thirds, the fourths as they are called, they are making more money than companies themselves, because they buy cheap milk and they make mozzarella out of it, they make certain cheeses and sell it under the table*.

## Discussion

The findings of the quantitative component indicate some attributes that, in their integration and comparison with the theory constructed in the qualitative analysis, allow a better understanding of the phenomena around trust and the reciprocity norms in the framework of udder health (see Table 5).

**Table 5:** Matrix for comparison of similarities and integration of qualitative and quantitative results.

Variable	Attributes	Theory (based on qualitative analysis)
Trust in veterinarians	90.3% trust Higher BTSCC for those who do not trust. higher CFU for those who do not trust.	Trust is the most important element of collective action. It depends on time, patience, charisma, and evidence or results. There is reciprocal trust in dairy farmers when they fully implement the recommendations of veterinary. It is based on theoretical grounds, experience, and respect. The poor labor conditions of veterinary promote employee turnover and the repetition of the trust-building cycle with the new veterinarian. Non-face-to-face veterinary care discourages trust in their recommendations. The high cost of drugs in an integral treatment affects trust between dairy farmers and veterinarians and the viability of recommendations.
Trust in other dairy farmers’ recommendations	71.3% trust Higher BTSCC for those who trust. Higher CFU for those who trust.	There are different groups; there are those who participate in collaborative groups and believe in their recommendations, as well as those who consider that the recommendations of other dairy farmers are malicious. Some consider the establishment of cooperative networks a generational issue, where older adults are willing to participate, and the youth is now more egocentric. Leaders are key players in promoting collective action.
Trust in workers	77.3% trust Higher BTSCC for those who do not trust. Higher CFU for those who do not trust.	Having workers is a problem for improving udder health. They lack commitment and have inappropriate milking and animal handling practices. Long and hard-working hours and poor labor conditions are detrimental to trust between the parties and udder health. Motivating workers with bonuses improves udder health.
Trust in udder health care support from collection companies.	80.6% trust Higher BTSCC for those who trust. Higher CFU for those who do not trust	Dairy farmers consider that the payment for CFU and BTSCC is very low and manipulated. Bonuses have been withdrawn by the collection companies, limiting producer trust and motivation. The supply of veterinary services offered by the collection company is deficient. The veterinary care service is aimed at reproduction, not udder health. Dairy farmers never receive directly from the laboratory the results of the analysis of the milk sold to a collection company. They only receive them on their payment slip, which generates mistrust.
Trust in ICA monitoring—udder health	58.8% do not trust Higher BTSCC for those who trust. Higher CFU for those who trust.	The ICA does not provide any kind of support to dairy farmers regarding udder health.
It is the government’s responsibility to promote regulation on udder health protection.	90.3% agree Higher BTSCC for those who agree. Higher CFU for those who agree.	Government actions that destabilize producer trust: The lack of government control over collection companies about milk payments undermines trust in the government. The milk sector lacks regulations that promote economic growth. Free trade agreements negatively affect dairy farmers in Colombia and, therefore investment in udder health. The government has not established policies to control the prices of concentrates and fertilizers. Due to the high cost of inputs, the government has not established producer support programs to address economic crises. Corruption in economic support programs for dairy farmers working in food production has deteriorated trust between dairy farmers and the government.
Trust in milk producer cooperatives as a strategy for collective action.	91.7% trust Higher BTSCC for those who do not trust. Higher CFU for those who do not trust.	Membership in a cooperative favors udder health through benefits that non-members do not have. Although some consider that there are no longer differences between members and non-members, except in the guarantee of milk purchase in case of an *enlechada*.
Trust that the laboratory is the best alternative for the choice of antibiotic.	94.4% trust Higher BTSCC for those who trust. Higher CFU for those who trust.	There are no independent laboratories in the region. Some dairy farmers are not willing to invest in mastitis diagnosis. This promotes the inappropriate use of antibiotics and distrust towards the collection companies. The delivery time of results considering the remoteness of laboratories and the difficulty of bringing the samples limits the appropriate decision-making in the diagnosis and treatment of mastitis.
The attitude of reciprocal cooperation in the appropriate use of antibiotics according to laboratory results	94.4% agree Higher BTSCC for those in disagree. Similar CFU between those in agreement and those in disagreement.	Dairy farmers accept recommendations from veterinarians and other actors regarding antibiotic choice without using the laboratory. The attitude disagrees with the behavior due to the lack of laboratory services in the area.
The attitude of reciprocal cooperation concerning not selling milk from cows with mastitis and antibiotics.	98.1% agree Higher BTSCC for those who agree. Higher CFU for those who agree	Although all dairy farmers recognize the impact that the sale of milk from cows with mastitis and antibiotics has on public health and express their attitude of not selling such milk to the informal trade, the complex economic situation in which they find themselves has led them to act inconsistently.

Trust is a reciprocal principle that is by nature evident in the relationships that dairy farmers establish with other actors in the dairy chain. Trust between producer and veterinarian in the context of udder health depends on communication processes and cultural recognition to achieve optimal results in udder health improvement measures [7]; we additionally found that time, patience, and charisma are critical in recognition and reciprocal communication, which is consistent with the mutualistic communication already reported. Recognition of dairy farmers’ knowledge and experience promotes collective action; it has been identified that when veterinarians use a mutualistic communication style rather than more traditional paternalistic communication, they appear to show greater adherence to advice [15]. In Sweden, the trust evidenced in adherence to advice was related to (I) trust in veterinarians, recommendations given, and the advisory process, (II) the feasibility of the recommendations, and (III) the perceived seriousness of the problem and the perceived need to implement the suggestions. For veterinarians to improve adherence to recommendations, they must focus their attention on the dairy farmers’ needs, priorities, goals, and motives, as well as on the dairy farmers’ perceptions of the effectiveness of individual preventive measures, understand the practical routines and costs of each farm and acknowledge dairy farmers’ expertise [6]. In our study, we found that the trust relationship between these two actors depends to a large extent on credibility, and this, in turn, is based on veterinarians’ theoretical capacity, experience, and respect for dairy farmers’ motivations and limitations to implementing recommendations; in turn, the positive results originating in their recommendations strengthen trust. Understanding the motivational factors that enable dairy farmers to make decisions is complex. There is an underlying attitude toward evidence-based veterinary practice and achieving satisfactory results, where veterinary recommendations must be supported by solid, objective, and up-to-date evidence [15]. At the same time, it has been found in North America that the lack of time and limited finances of dairy farmers represent barriers to following recommendations regarding the use of antibiotics [16]. In the North of Antioquia, the high cost of medications is a limiting factor for following recommendations despite the trust built.

It has been found that dairy farmers’ trust profiles are highly variable concerning their trust in actors that represent sources of information required for decision-making. In Costa Rica, they found that the family is more important in detecting problems and seeking opinions, while the role of technical advisors is more important in solving problems and seeking new practices. In third place is the staff working on the farm being important in the detection of the issues, showing a process of trust between producer and workers [17], which contrasts with the results of our study, where the family is considered important. However, the statistics show that only 11.3% of the children decide to work on the farm, which represents a near future without generational replacement in primary milk production. In turn, in the north of Antioquia, workers behave differently- They show a lack of commitment and unwillingness to report situations that put the health of the udder and the quality of the milk at risk. This attitude seems to be related to their labor conditions and the absence of economic incentives. Other dairy farmers were less reliable sources of information for decision-making. However, they are considered by dairy farmers as moderately reliable in the search for new practices. Sutherland et al. (1996), quoted by Solano, 2003, found that dairy farmers can be key actors of reference in a community of dairy farmers, but with certain conditions based on results, where distrust of the self-reported performance of other dairy farmers prevails; while their follow-up was perceived as of great interest [17], this behavior is consistent with what was observed and discussed with dairy farmers in the north of Antioquia.

Alterations in udder health can affect food safety about the food safety of raw milk. An organizational theory proposes that one way to improve food safety in dairy products is through smallholder membership in producer organizations or cooperatives.

This theory proposes a framework of social control, process control, and output control [18]. The first type of control refers to relationships of trust and reciprocity norms based on collective action. Social control is constituted by social mechanisms that directly or indirectly influence the behavior of other dairy farmers who have a common interest, and by social capital, in terms of trust, which is greater between dairy farmers than trust in the dairy farmer-merchant relationship [19]. Dairy farmers benefit from cooperative services related to food safety, training, milk collection, monitoring and evaluation of food safety measures, and provision of milk storage equipment [18]. It is considered that for small farmers to be competitive, institutional arrangements are required. Cooperativism through producer organizations has been found to influence safe food production behaviors among dairy farmers and promote the adoption of food safety measures related to milk storage and milking area; in addition, social incentives to improve food safety adoption among dairy farmers are relevant, even when price incentives are absent [5]. In the north of Antioquia, it was found that being associated with a dairy farmers’ cooperative organization promotes udder health and food safety since such membership allows access to health care services, discounts in the purchase of inputs, and professional advice; however, there are differences between the benefits offered among the different cooperatives and some dairy farmers state that due to their bad experience they have found few differences between being or not being a member of a cooperative.

The trust of northern Antioquia dairy farmers in the government is deteriorating because of the lack of strong policy interventions to improve the competitiveness of the Colombian dairy sector in productivity, product quality, and foreign trade, and in turn by its inability to control the prices of inputs required for production. These situations, which condition dairy farmers’ economy, limit their capacity to invest in mastitis control and prevention, in the promotion of udder health, and investment in the technification of production systems. In this sense, Asoleche warned in 2019 about the risk of free trade agreements for national dairy farmers due to inequitable negotiations. With the United States, imports that began in 2012 were negotiated at 9,570 tons of dairy products and that in 2019 totaled 18,650 tons, all without tariffs and with an annual growth rate of 10%, where once the agreed amount per dairy product is exceeded, imports can continue paying a tariff that gradually decreases reaching 0% in 2026; likewise, imports arranged with the European Union will be tariff-free in 2028 [20]. It is estimated that for each ton of milk, 8,000 liters are no longer purchased from domestic dairy farmers due to their inability to compete in terms of quality and price [20]. The European Union produces milk with high-quality standards and its dairy farmers receive subsidies and internal aid, a situation that contrasts with Colombia, where the government does not grant such economic benefits to primary farmers [21]. Free trade agreements have directly and negatively affected the purchase prices of national dairy farmers, which has a direct impact on their capacity to invest in improving productivity and udder health and on their quality of life.

In this study, it was found that the absence of government reaction to the control of input prices, mainly concentrates, and the control of milk prices has led to the demotivation of all dairy farmers, distrust towards the government, and bankruptcy of many of them in recent years. This situation differs from the European reality, where changes in feed prices in the United States are not an additional risk for the profitability of EU dairy farmers, since milk price and feed costs are positively correlated and, therefore, the margin between milk price and feed price is relatively stable, being a public policy of state protection [22]. The risks in the profitability of milk production related to the price of concentrate vary with the level of specialization of the farm. For example, the income of an extensive pasture-based system may depend less on changes in the price of concentrate than the income of a high-performance milking system, which is intensively fed with concentrates [23]. In the north of Antioquia, production systems are based on pasture and concentrate consumption, which represents for the Colombian case the need to make production systems more technical and government intervention with economic protectionism and udder health policies; as Jeffrey Fajardo, executive president of Asoleche stated in 2019, "Definitely, the dairy sector requires an urgent reformulation of all the public policy mechanisms that have tried to isolate it from the world market phenomenon and, in turn, to move towards a much more competitive scheme, that is, progressively and increasingly guided by the market" [20].

As a measure to mitigate the impact of the increase in raw milk producer inputs in Colombia since the end of 2020, the Colombian Ministry of Agriculture and Rural Development decreed in Resolution 0290 of 2021 a 7% increase in the value of the gram of total solids, protein, and fat for the payment of milk to dairy farmers, as well as an increase in allowances and penalties per CFU. This decision was taken by the National Dairy Council (Consejo Nacional Lácteo) because of the constant increases in the prices of protein and energy inputs to produce animal feed, an increase of 35 to 51% in the price of balanced feed, increases in fertilizers between 24 and 51% and up to 40% in production costs [24]. However, dairy farmers and veterinarians consider that this adjustment is not enough, and comprehensive interventions are required to address the problem.

For several years, the payment of milk has represented discomfort among primary farmers due to the economic conditions described above and the low annual increase in the payment of milk, together with the results of the indicators of hygienic, healthy, and compositional quality with which milk is paid, which dairy farmers consider are manipulated by the dairy companies, assisted by the lack of state control over them. In compliance with Conpes document 3675 of 2010, the Ministry of Agriculture and Rural Development established in 2012 an articulated work with the Colombian Corporation for Agricultural Research (Agrosavia), as the coordinating entity of the subsystem of evaluation, verification, and ordering of laboratories for the analysis and payment for milk quality, to contribute to the strengthening of laboratories, which allows their accreditation in the standard NTC-ISO/IEC 17025:2005 [25]. This standard only defines the criteria for the qualification and certification of milk laboratories, but does not address the processes of control and guarantee results directly to dairy farmers, which are necessary, given that the government has left the responsibility of monitoring the quality of farmers’ milk actors with a conflict of interest such as the collection companies who have their own laboratory or subcontract with an external certified laboratory, which only delivers the results to them and not to the dairy farmers. Dairy farmers and veterinarians in the municipalities studied indicate that the results of the analysis of counter-samples evaluated by dairy farmers or the differential payment to dairy farmers who share tanks are evidence of irregularities in the payment of milk by some collection companies and the lack of state control.

Governments in other countries have intervened in udder health problems in a variety of ways. The United Kingdom has supported dairy farmers with subsidies and domestic support such as education and technification of production systems [21]. Additionally, they have maintained a positive correlation between the price of feed for cows and the price of milk [22]. Nicaragua has addressed udder health through pasture improvement and training on the technification of pasture production [26]. In Australia, efforts are being made to strengthen commitments among the actors in the dairy chain [27]. European countries such as Great Britain, Netherlands, Denmark, Belgium, Germany have successfully implemented campaigns and programs around the concept of udder health, from the technification of production systems, animal welfare, the formulation of health plans and the use of antibiotics and they have acknowledged that despite the technological advances that have facilitated the production process, and health planning, the active and voluntary participation of dairy farmers is required for the implementation of new methodologies [28]. Based on the above, analysis of the cultural diversity of dairy farmers and their attitudes that define behaviors and, in turn, the collaborative networks and norms that influence decision-making for udder health improvement has been considered paramount in their campaigns and programs [28]. The European experience has taught that for knowledge to be applied, communication with producers is as important as the technical content of the message, and it is necessary to consider that today’s dairy farmers are not comparable to those of decades ago [28]. For example, in the Netherlands, a comprehensive udder health policy has been implemented where knowledge and culture in terms of attitudes that define behaviors by the dairy farmer is the basis for the design of programs and the evaluation of success, that is, they focus on sociocultural transformation and collective action that originates in the construction of trust [7, 8].

According to our results, reasons such as the remoteness of the laboratories, the time in the delivery of results, and the attitude towards not investing in mastitis diagnosis and antibiogram, promote the inappropriate use of antibiotics in the face of clinical mastitis or the implementation of a drying therapy. These results contrast with the attitude towards clinical and milk laboratory use in Denmark where general dry cow therapy is not allowed in the country, and the BTSCC result represents one of the main indicators used to select cows for laboratory evaluation through bacterial culture or PCR, which has allowed successful antibiotic-based selective drying-off treatment based on antibiogram results [29]. Resistant bacteria transferred from animals to humans are a public health concern. In the USA, barriers to appropriate antibiotic treatment were found to be lack of time and limited finances, as well as only 45% of dairy farmers, make use of the laboratory (culture and antibiogram), 25% decide to treat mastitis with antibiotics based on field mastitis tests, and all consider the presentation of signs to be a criterion for determining the need to use an antibiotic against mastitis [16]. Concurring with trust in the laboratory and the feasibility of its use to improve udder health conditions described in the north of Antioquia, a study in Canada describes that cost, the feasibility of sample collection during milking routines, and the extensive processing of samples are important obstacles for the diagnosis and epidemiological study of mastitis [30].

The study found a common involvement of untrained actors in the choice of antibiotics for mastitis treatment. In agreement, in Tanzania, they also found that dairy farmers mainly rely on the recommendations of private veterinarians regarding the use of antibiotics and that other professionals, pharmaceutical vendors, or technicians also participate in the decision-making process [31].

Although antimicrobials are used for therapeutic purposes to improve animal health, welfare, and productivity [32], the consumption of milk from cows on antimicrobial therapy without withdrawal times can affect the animal and consumers’ health, and contaminate the environment, since inappropriate use of antibiotics can favor bacterial resistance [33, 34]. In our study, it was found that despite a good attitude of dairy farmers not to take milk from cows with mastitis and under antibiotic treatment to the informal trade, there are conditions, mainly economic, that force them to do so. According to the Colombian Association of Dairy Farmers, informality is conditioned by the low price paid to dairy farmers, the inability of the formal industry to buy the entire production, the difficult access to farms, the lack of interest of dairy farmers to pay taxes, and the inability to improve milk quality [35]. Informal trade has been poorly documented in scientific studies. In Tanzania, dairy farmers reported frequent use of veterinary medication such as oxytetracyclines, penicillin, and streptomycin on cows in production. Most dairy farmers did not discard milk during or after treatment, and less than half of the dairy farmers boiled milk, although the sale of fermented milk was common. In addition to this behavior, cattle management was characterized by low levels of biosecurity, inadequate hygiene, and disease control practices [31].

In summary, Colombia has long studied mastitis from a biomedical approach [36–41], but little progress has been made in the concept of udder health [14]. Udder health is a complex phenomenon mediated by many factors, including sociocultural and political factors. Trust is one of the categories of analysis that allows understanding sociocultural and political aspects of udder health. In order to understand the relationships of trust between dairy farmers and other actors in the dairy chain, it must be assumed that udder health is a common good and that its problems affect all the actors or links in the dairy chain. Interventions should therefore be based on this theory and be based on collective action [2]. The study of trust between these actors allows us to understand why mastitis control interventions are not as successful as expected. In this sense, the dairy farmer-veterinarian relationship is the most important relationship for improving udder health conditions [6]. This relationship depends on trust, and trust in turn depends on the way they relate to each other for decision-making in the control and prevention of mastitis [6]. For example, communication strategy [15], farmer participation in health planning [42], consideration of the interventions’ feasibility [6], farmer motivations [15], and clear theoretical and scientific support for the farmer, among others [16].

In the case of other actors, trust also has outcomes on production and udder health. Farmers’ trust in workers depends on working conditions, their education, motivation with bonuses, and their interest in detecting and reporting situations that affect the udder [17]. Trust among farmers has deteriorated when recommendations are not successful. This fact shows the complexity of mastitis and the need for professional advice [17].

The participation of dairy farmers in associations and cooperatives establishes a type of horizontal relationship in which there are relationships of trust and reciprocity in a context of social control and common benefits, i.e., collective actions [18]. Cooperative dairy farmers have shown that through access to health services, training, and commitment to milk production, milk quality, and food security are improved [18].

Although the relationship between the government and dairy farmers does not seem to be direct. Trust between these two actors is mediated by government actions in relation to input price control, milk price control [22], free trade agreements for milk, milk derivatives, and dairy products, the actions of its institutions such as the ICA and INVIMA [20, 21], aid and subsidies [21] and the design of programs that consider the culture of dairy farmers and collective action [7, 8]. These elements constitute commercial, economic, and political factors that have deteriorated farmers’ confidence towards the government with results such as: 1. Low investment capacity in control and prevention and the technification of production systems [21] 2. Affections on the hygienic and sanitary quality of milk [28].

Any udder health program should consider not only the technical aspects, but also work on the specific problems of udder health, the actors that can participate in their solution, the trust building for efficient collective actions based on the characterization described above.

## Conclusions

Trust Representation, Socio-cultural Factors, Economic and Commercial Factors, Labor Conditions, Clinical, and Milk Laboratory, and Reciprocity Norms constitute the categories of analysis in theorizing about trust and udder health. The integrated analysis of quantitative and qualitative results illustrates the complexity of the trust phenomena that are established between dairy farmers and other actors in the dairy chain and that determine udder health care and milk production. Each of the theoretical and emerging categories in this study account for actors, attitudes, behaviors, relationships between actors, and norms that allow understanding that trust between dairy farmers and other actors in the dairy chain to face udder health problems and milk production. Trust depends on technical processes, individual and collective human attitudes, and behaviors, service supply, political, regulatory, and economic determinants, the latter being pivotal in decision-making to invest in mastitis control and udder health care. Udder health problems affect all links in the dairy chain. Therefore, udder health is a common good. As a common good, the solution to the problems that affect it does not depend on a single individual, but on collective actions. These collective actions are based on trust. Hence the importance of understanding how trust relationships arise, what they depend on and how they can be strengthened.

### Study limitations

This study was conducted over a limited period and therefore presents an analysis of dairy farmers’ experiences of the trust-udder health relationship up to mid-year 2021. The national government restructured laws related to milk payment and input costs after fieldwork was conducted and during the analysis of information and writing of articles. Contrary to expected, after analyzing all the categories identified, we can conclude that the proposed measures are insufficient. Time and funding limitations did not allow us to include other dairy chain actors in the study, who would have enriched the analysis of the information. There are no studies in Colombia on the object of study we proposed, so our findings were contrasted with international literature. Despite these limitations, the analysis of the categories that emerged in the study was deepened. In the quantitative component, the sample was by convenience. Accordingly, the findings could not be extrapolated to the rest of the population. In the qualitative component, transferability criteria were applied to select participants representing the diversity of the population. The low participation of female participants in this study is a portrait of the distribution of men and women engaged in dairy production in the region. According to our analysis, gender has no impact on udder health.

### Scope of future studies

The theoretical basis developed in this study, together with the theory that emerges from the studies of networks, institutions, attitudes, behaviors and public health in the framework of the macro project on cultural processes, social capital, and udder health will serve as input to propose, through work with the actors of the dairy chain and the government, priority guidelines for the restructuring of public policies to udder health, and milk production and commercialization.

## Supporting information

S1 Data This file contains the database of the quantitative component. (XLSX)

S1 File This file contains the topics included in the interviews. (DOCX)
