# Supplementary material for: Trust and reciprocity norms in the analysis of social capital related to udder health. A mixed methods approach with dairy farmers and veterinarians from the north of Antioquia
Source: PLoS One. 2023 Nov 17;18(11):e0277857. doi: 10.1371/journal.pone.0277857 (PMC10655989; doi:10.1371/journal.pone.0277857)
Supplement: S1 File — (DOCX) [file pone.0277857.s002.docx]

**Appendix 7. Trust and Udder Health: An Interview**

| **Category** | **Topic** |
| --- | --- |
| Trust | Representation  Exploring udder health  Improving milk payment (SCC and RSC)  Other actors involved  Social influences  Cooperativism - reciprocal cooperation  Leadership - social references - local collective actions  Actions to strengthen trust with other participants in the dairy chain  Collective action to improve udder health  Actions by other actors to improve udder health  Sociocultural factors that promote and limit trust  Economic and commercial factors affecting trust |
